# An amino acid-electrolyte beverage may increase cellular rehydration relative to carbohydrate-electrolyte and flavored water beverages

**DOI:** 10.1186/1475-2891-13-47

**Published:** 2014-05-26

**Authors:** Chih-Yin Tai, Jordan M Joy, Paul H Falcone, Laura R Carson, Matt M Mosman, Justen L Straight, Susie L Oury, Carlos Mendez, Nick J Loveridge, Michael P Kim, Jordan R Moon

**Affiliations:** 1 MusclePharm Sports Science Institute, MusclePharm Corp., 4721 Ironton St., Building A, Denver, CO 80239, USA; 2 University of Nebraska, Lincoln, NE, USA; 3 Metropolitan State University of Denver, Denver, CO, USA; 4 University of Northern Colorado, Greeley, CO, USA; 5 Department of Sports Exercise Science, United States Sports Academy, Daphne, AL, USA

**Keywords:** Rehydration, Fluid retention, Amino acid, Carbohydrate, Electrolyte, Hydration, Recovery

## Abstract

**Background:**

In cases of dehydration exceeding a 2% loss of body weight, athletic performance can be significantly compromised. Carbohydrate and/or electrolyte containing beverages have been effective for rehydration and recovery of performance, yet amino acid containing beverages remain unexamined. Therefore, the purpose of this study is to compare the rehydration capabilities of an electrolyte-carbohydrate (EC), electrolyte-branched chain amino acid (EA), and flavored water (FW) beverages.

**Methods:**

Twenty men (n = 10; 26.7 ± 4.8 years; 174.3 ± 6.4 cm; 74.2 ± 10.9 kg) and women (n = 10; 27.1 ± 4.7 years; 175.3 ± 7.9 cm; 71.0 ± 6.5 kg) participated in this crossover study. For each trial, subjects were dehydrated, provided one of three random beverages, and monitored for the following three hours. Measurements were collected prior to and immediately after dehydration and 4 hours after dehydration (3 hours after rehydration) (AE = −2.5 ± 0.55%; CE = −2.2 ± 0.43%; FW = −2.5 ± 0.62%). Measurements collected at each time point were urine volume, urine specific gravity, drink volume, and fluid retention.

**Results:**

No significant differences (p > 0.05) existed between beverages for urine volume, drink volume, or fluid retention for any time-point. Treatment x time interactions existed for urine specific gravity (USG) (p < 0.05). Post hoc analysis revealed differences occurred between the FW and EA beverages (p = 0.003) and between the EC and EA beverages (p = 0.007) at 4 hours after rehydration. Wherein, EA USG returned to baseline at 4 hours post-dehydration (mean difference from pre to 4 hours post-dehydration = -0.0002; p > 0.05) while both EC (-0.0067) and FW (-0.0051) continued to produce dilute urine and failed to return to baseline at the same time-point (p < 0.05).

**Conclusion:**

Because no differences existed for fluid retention, urine or drink volume at any time point, yet USG returned to baseline during the EA trial, an EA supplement may enhance cellular rehydration rate compared to an EC or FW beverage in healthy men and women after acute dehydration of around 2% body mass loss.

## Background

In conditions of elevated body temperature, the body can dissipate heat through either convection, conduction, radiation, or evaporation. However as temperature rises, the effectiveness of convection and conduction decreases and radiation becomes insignificant [1,2]. This creates a concomitant increase in dissipation through evaporation, accounting for up to 98% of cooling [1]. Thus, evaporation of sweat is the body’s primary mechanism for decreasing body temperature. Without replacement of fluids, an athlete can quickly become dehydrated during training or competition.

Robust fluid loss (5-10% of body weight) can be well tolerated by healthy individuals at rest [3], yet a 2-3% fluid loss impairs exercise performance [3-11]. In a study by Armstrong et al. [4], competitive runners were tested in time trials of varying distances as well as time to exhaustion under conditions of hydration and dehydration in a crossover design. Dehydrated athletes took longer to complete each time trial and experienced a decreased time to exhaustion. In a hot, humid environment, performance decrements can be further exacerbated by dehydration [12,13]. While athletes may adequately recover fluids lost with multiple days separating exercise sessions, those who partake in practice or competition daily or twice-per-day must utilize strategies to recover fluids lost as soon as possible after an exercise bout.

Water may typically be used by athletes to rehydrate, yet nearly every rehydration trial has determined water to be inadequate for rapid recovery of lost body fluids [14-20]. This is persistent even when high volumes of water are consumed due to the concomitant decrease in sodium concentration creating a hyponatremic state. However, simply adding sodium to water increases rehydration and better restores exercise performance [21], and the same observations are reported when adding sodium to milk [22] and coconut water [20]. The importance of sodium for rehydration is well established [23,24], yet evidence supporting potassium and carbohydrate are less robust.

Sodium is the major electrolyte of the extracellular space and is the primary ion lost in sweat [25], yet potassium is the primary intracellular electrolyte and has also indicated efficacy in rehydration [26,27]. Maughan and colleagues [28] have demonstrated equal fluid retention after consumption of sodium or potassium containing beverages administered after exercise induced dehydration. While supplementation with carbohydrates can directly benefit performance [18], they also assist in the rehydration process, though primarily through increased palatability [29,30]. In a similar fashion, proteins further increase rehydration when added to a carbohydrate- electrolyte (CE) beverage. Seifert et al. [16] compared the rehydration capabilities of a CE, protein-CE, and plain water beverage following a dehydration protocol. They observed that the addition of protein to a CE beverage resulted in greater increases in fluid retention and plasma volume compared to the CE and plain water beverages. However in addition to greater protein content, there was also a greater presence of electrolytes, notably 44% more potassium than the CE beverage. Similar findings have been noted when examining the protein containing beverage milk, which also has a greater presence of electrolytes [19,22]. Whether the increased fluid retention in these studies are due to additional protein or electrolytes remains ambiguous. However, protein can stimulate muscle protein synthesis and aid in glycogen resynthesis when carbohydrate intake is inadequate [31]. Thus while sodium is an important component of a rehydration beverage, the importance of potassium and other solutes, such as carbohydrate and protein, is not to be overlooked.

While protein is capable of increasing muscle protein synthesis, it is primarily the branched chain amino acids (BCAA) which drive this process, namely leucine [32,33]. Therefore, it is conceivable that if the addition of protein to a CE beverage may enhance rates of rehydration, the addition of its constituents, such as BCAAs, may enhance rehydration as well. In any event, they would surely benefit recovering muscle tissue and may be recommended for post-exercise consumption. However, the effects of BCAA’s on rehydration remains to be investigated. Additionally, previous research has administered test beverages in very large quantities, which are not practical for most athletes. Therefore, the purpose of this study was to compare the rehydrating effects of a BCAA-electrolyte (AE) beverage, CE, and flavored water (FW) control on exercise-induced dehydration and also to determine if a modest, more practical amount of these beverages in combination with water to compose the additional fluid requirement would provide adequate rehydration. We hypothesized the AE and CE beverages would rehydrate similarly, yet they would rehydrate to a greater extent compared to the FW beverage.

## Methods

### Subjects

Twenty men (n = 10; 26.7 ± 4.8 years; 174.3 ± 6.4 cm; 74.2 ± 10.9 kg) and women (n = 10; 27.1 ± 4.7 years; 175.3 ± 7.9 cm; 71.0 ± 6.5 kg) participated in this study. Each subject was required to have a minimum of one year endurance and resistance training experience, be free of any cardiovascular, metabolic, renal, hepatic, musculoskeletal, or any other disorder which may affect study outcomes, be free of any medication, supplements, or pharmaceutical which may influence measurements. Prior to testing, the study was approved by the MusclePharm Sports Science Institute Institutional Review Board and all subjects provided written informed consent to participate in this study.

### Experimental design

In a double blind design, subjects participated in each of three beverage trials in a randomized order. Each trial was separated by a washout period of at least 7 days. The contents and volume of drink consumed for each trial can be found in Tables 1 and 2. Baseline measurements were collected individually for each trial then repeated immediately and 4 hours post-dehydration. Following baseline measurements, subjects performed the dehydration and rehydration protocols. The rehydration period occurred during the hour immediately following post-dehydration measurements. All trials were conducted in the morning, and subjects were instructed to refrain from exercise for 24 hours, solid food consumption for 8 hours, and water consumption for 1 hour prior to each trial. Each subject’s hydration status was assessed upon arrival by urine specific gravity (USG). In the event USG was greater than 1.025, the subject was provided with 500 mL water in accordance with ACSM recommendations [34] to ensure all subjects began each trial in a euhydrated state. This occurred on only two occasions.

**Table 1:** Composition and amount of test beverage administered

	**AE**	**CE**	**FW**
Test Volume (mL)	772.5 ± 93.17	774.7 ± 100.9	770.8 ± 96.88
Calcium (mg)	96.2 ± 11.31	7.9 ± 1.00	.2 ± 0.02
Phosphorous (mg)	116.3 ± 13.67	78.6 ± 9.98	.2 ± 0.02
Magnesium (mg)	400.8 ± 47.12	0	0
Sodium (mg)	80.2 ± 9.43	311.7 ± 39.57	1.2 ± 0.14
Potassium (mg)	200.5 ± 23.57	120.5 ± 15.30	121.3 ± 14.86
BCAA (g)	6.0 ± 0.71	0	0
Carbohydrate (g)	2.0 ± 0.24	52.4 ± 6.65	.4 ± 0.05
Calorie (kcal)	10.0 ± 1.18	207.0 ± 26.27	8.9 ± 1.09

The total amount of solute present is provided. Data are presented as mean ± SD.

**Table 2:** Dependent variables between treatments

	**AE**	**CE**	**FW**
Total drink volume (mL)	2292.25 ± 457.99	2279.26 ± 483.63	2360.44 ± 519.75
Test drink volume (mL)	772.46 ± 93.17	774.73 ± 100.9	770.83 ± 96.88
Water drink volume (mL)	1519.79 ± 388.04	1504.52 ± 428.92	1589.62 ± 476.92
Total urine volume (mL)	1295.5 ± 457.56	1348.25 ± 450.94	1363.25 ± 520.44
Fluid retention (%)	43.5 ± 17.1	40.8 ± 18.1	42.2 ± 14.3

Data are presented as mean ± SD. Fluid retention was calculated as: (Total Drink Volume - Total Urine Volume) / Total Drink Volume.

### Dehydration and rehydration

Dehydration was achieved through a combination of exercise and sauna. To begin the dehydration protocol, each subject ran on a treadmill for 30 minutes at 80% of their estimated max heart rate (206.9 - 0.67 × age). Afterwards, subjects sat in a sauna at 70°C in 15 minute intervals such that about a 2% body weight loss was attained (AE = −2.5 ± 0.55%; CE = −2.2 ± 0.43%; FW = −2.5 ± 0.62%). Between sauna intervals, 2 minutes were dedicated to collecting subjects’ nude, dry body weight. During rehydration, subjects were administered equal volumes of the AE (Amino1™, MusclePharm Corp.), CE (Gatorade™, Pepsi Corp.), or FW (Crystal Light™, Kraft Foods Corp.) beverage immediately after dehydration testing. Initially, subjects were provided with ~30-35% of their total drink volume as test beverage. The amount of test beverage was determined primarily by the AE condition. Wherein, 0.25 g of amino acids per kg baseline body weight was the target for the AE condition, as this has been shown to positively influence nitrogen balance [35]. It also produced reasonable doses for all test beverages. After delivery of the test beverage, the remaining volume was equally divided into 3 doses of water provided over the final three 15 minute intervals such that total fluid ingestion occurred over a one hour period immediately after dehydration testing. The total volume of beverage administered was equal to 150% of fluid lost during dehydration [36]. Thus, the volume of water was determined as the calculated total volume minus the calculated test volume. Subjects were required to consume the entire volume of fluid in the allotted 1 hour rehydration period. Beverage composition can be found in Table 1.

### Measurements

Measurements for all trials were taken prior to, immediately following, and 4 hours following the dehydration protocol. At baseline and immediately post-dehydration, nude body weight and USG were assessed and subjects were instructed to empty their bladder completely. Subjects were instructed to report to the laboratory without voiding and upon arrival to void as much as possible into a collection jug. From this, USG was measured using a handheld refractometer (Atago, Tokyo, Japan). At 4 hours post-dehydration, the aforementioned variables were reassessed as well as total urine volume over the 4 hour rehydration period and fluid retention was calculated as total drink volume minus 4 hour total urine volume divided by total drink volume.

### Statistics

All data were analyzed using SPSS software (Version 20.00, SPSS Inc., Chicago, Illinois, USA). A 3 × 3 repeated measures ANOVA was used to determine treatment by time interactions for USG and urine volume. A one-way ANOVA was used to determine differences in drink volume and fluid retention. A Bonferroni post-hoc analysis was used to locate differences.

## Results

**Figure 1:**
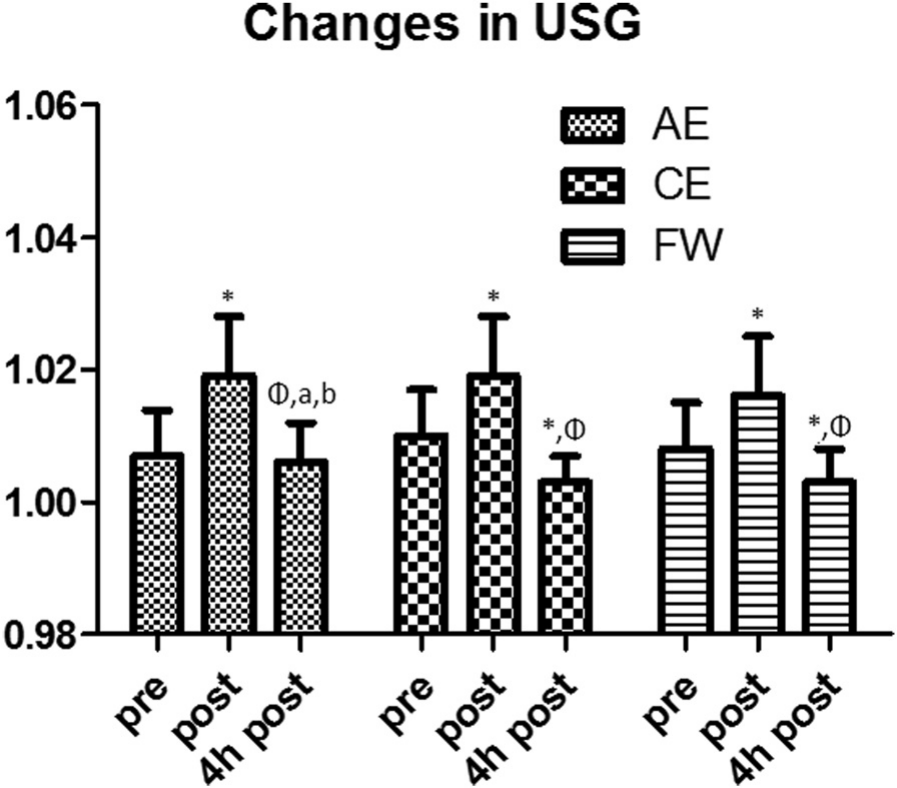
**Changes in USG.** * indicates significantly different from pre (p < 0.05). Φ indicates significantly different from post. a indicates significantly different from CE. b indicates significantly different from FW. Pre represents baseline measures, post represents immediately post-dehydration, and 4 h post represents 4 hours post-dehydration.

No significant differences (p > 0.05) existed between beverages for percent body weight loss, drink volume (test or total), urine volume, or fluid retention for any time point (Table 2). Treatment x time interactions were observed for USG (p < 0.05; F = 0.783) (Figure 1). Post hoc analysis revealed differences occurred between the EA and FW beverages (p = 0.003) and between the EA and EC beverages (p = 0.007) at 4 hours after dehydration.

## Discussion

The results of this study both partially agreed and disagreed with our hypotheses. The AE and CE beverages rehydrated about equally; however, they were also equal to the FW beverage. The purpose of this investigation was to compare the rehydration capabilities of AE, CE, and FW beverages. The primary finding of this study was USG for the AE condition at 4 hours returned to baseline following dehydration while CE and FW trials resulted in more diluted urine (lower USG values). Additionally, there were no significant interactions for percent body weight loss, urine volume, drink volume, or fluid retention for any condition. On the basis of these findings, it would appear that all conditions were equally capable of rehydration. However, because an equal fluid retention was observed between all conditions, yet USG did not show the same decremented pattern (dilution at 4 hours) as CE or FW, it appears that the AE beverage increased intracellular hydration more favorably than either CE or FW since the urine was not as diluted at 4 hours in the AE trial with equal urine volume (Table 2). Thus at 4 hours post-dehydration, subjects experienced the same USG as pre-testing, while at the same time-point, CE and FW trials resulted in significantly lower USG values compared to pre-testing which suggests increased diuresis through urine and less cellular retention. Furthermore, based on the USG results, it is hypothesized that if urine volumes were collected beyond 4 hours post-dehydration (3 hours post rehydration) that both CE and FW would have resulted in greater urine volumes compared to AE and USG would eventually return to baseline. Since there was an apparent increase in water in the bladder at 4 hours post-dehydration for CE and FW and USG had not returned to baseline, the body may continue to shed water until USG returned to baseline. This hypothesis can be supported further by the reduced urine volume in the AE treatment compared to CE and FW as there was a trend at 4 hours post-dehydration for AE to retain more water (Table 2).

No significant interactions were observed between conditions for urine volume, drink volume, or fluid retention at any time point. These results are contradictory to those previously reported [16,19,28]. In each of these studies, greater electrolyte content produced greater fluid retention. A greater fluid retention was expected for the AE and CE conditions compared to the FW. However, it is possible that the 121.3 mg of potassium in the FW condition was sufficient to produce insignificant results compared to the greater overall sodium and potassium content of the AE and CE conditions. In agreement with the present results, Mitchell and colleagues [37] examined 4 different relationships of drink volume and sodium concentration, and it was apparent that drink volume had a greater influence on rehydration compared to sodium content. However, it is important to note that the low sodium condition contained half of the sodium of the high sodium conditions, and the beverages were not devoid of electrolytes. From these results as well as our own, there is evidence for a limited amount of electrolyte present in the rehydration beverage to be capable of modest rehydration.

While sodium is the primary electrolyte of the extracellular space and has a pronounced role in rehydration [25], potassium, the primary electrolyte of the intracellular space, has demonstrated similar rehydration capabilities [26,27]. Yawata [26] examined the replacement of body fluids in each fluid compartment for sodium and potassium in dehydrated rats. It was observed that sodium preferentially rehydrated the extracellular space and potassium rehydrated the intracellular space. It was also noted that rehydration of the intracellular space took precedence over rehydration of the extracellular space, potentially indicating the relative importance of rehydrating the intracellular space. In the present study, the absolute amount of potassium consumed was similar between the CE and FW conditions. However, the AE condition consumed nearly twice as much as the CE and FW conditions, 643.6 mg vs 354.5 mg and 379.3 mg, respectively. The current findings indicated less diluted urine 4 hours following dehydration and a USG not significantly different than pre-testing for the AE condition only. Considering this and the absence of differences observed in percent body weight loss, urine volume, drink volume, or fluid retention, it appears that the AE beverage favored rehydration of the intracellular space. This is in agreement with Nielsen et al. [27] who observed preferential rehydration of the intracellular space with potassium compared to sodium. Additionally, the aforementioned researchers discovered that sodium increases plasma volume to a greater extent than potassium, which may also explain the differences observed for USG in the present study.

In addition to potassium differences, the AE beverage contains BCAAs as well as taurine and coconut water powder. While this may be the first study to investigate BCAAs effects on hydration, taurine has been previously examined. Muscles expel taurine during contraction [38,39]. Cuisinier et al. [40] examined plasma taurine levels during exercise in both hydrated and dehydrated states, observing a 32% greater increase in plasma taurine levels in the hydrated condition. This led researchers to conclude taurine is released due to an osmoregulatory process and to be taken up by other cells which have a role in osmotic regulation. Coconut water has demonstrated efficacy over water yet with mixed results. It has been observed as equal to a CE beverage for rehydration [20,41]. In contrast, Saat et al. [17] have reported greater rehydration effects with a CE beverage, despite the coconut water condition consuming more electrolytes, yet it has been shown as well that the addition of sodium to coconut water increases rehydration [20].

From the present results, it is also possible that carbohydrates were not necessary for rehydration, as no differences between the AE, CE, and FW beverages were observed for fluid retention. Few researchers have examined carbohydrates for rehydration in the absence of electrolytes. However, lambert et al. [42] conducted such comparison and found no significant differences between beverages containing or not containing carbohydrates, concluding that the benefit of carbohydrates is glycogen replenishment and taste, not rehydration. In agreement, varying glucose concentrations had no effect on rehydration when controlling for electrolyte content [43].

Unique to this study, subjects were rehydrated initially with the test beverages using a practical dose and followed up with water to replace 150% of lost body weight, which may more adequately simulate athletes’ common practice. Wherein, an athlete may drink one bottle (24 fl oz; ~26 fl oz were used in this study) of a recovery drink then drink water the remainder of the day or several hours thereafter. In this practical model, there were no observed differences in rehydration capability between all test beverages, yet the AE and CE beverages were anticipated to rehydrate more adequately than FW, as hypothesized. Therefore, it may be necessary for the dehydrated athlete to drink uncommonly more of an AE or CE beverage than they would expect to achieve the results observed in past literature [16,19,28]. However, the practicality of ingesting such large amounts of a CE beverage warrants concern as high doses of simple sugars may not be advantageous to all athletes.

The present study provides evidence for electrolytes being the primary determinant of rehydration when ingesting practical/real world volumes, regardless of calorie or macronutrient content. Future research is needed to examine BCAAs effects on rehydration without electrolytes or other confounding factors compared to carbohydrate and/or electrolytes. It may also be of interest to examine recovery of performance following dehydration using an AE compared to a CE beverage.

## Conclusion

Overall, there appears to be a potential benefit of a BCAA and/or potassium containing beverage on intracellular hydration. However, it is not clear from the present study which of the two solutes are driving this process. Alternatively, the delivery method of the beverages or the amount of test beverage could have been insufficient to produce anticipated results as reported in past investigations. Presently, we can conclude an EA and potassium supplement enhances the speed of cellular rehydration compared to an EC or FW beverage in healthy men and women after acute dehydration of about 2% body mass loss, as the AE urine returned to baseline concentration prior to CE or FW, yet no differences existed for urine volume, drink volume, or fluid retention. Practical amounts of all beverages used in the current investigation with a total fluid volume equal to 150% of water lost during dehydration all appear to rehydrate. However, baseline urine concentration (USG) was achieved with the same fluid loss at 4 hours post-dehydration for AE only compared to CE and FW. Future studies should look at similar products over a longer rehydration (post-dehydration) period to assess the total volume retained from CE and FW beverages as the current study suggests more than 3 hours post-rehydration is needed for CE and FW to return to baseline USG values and a greater amount of urine volume (less retention) may be observed compared to a AE beverage similar to the one used in the current investigation.

## Abbreviations

BCAA: Branched chain amino acids; EA: Electrolyte amino acid; EC: Electrolyte carbohydrate; FW: Flavored water; USG: Urine specific gravity.

## Competing interests

CT, JJ, PF, LC, MM, NL, MK, and JM are employees of the funding source, MusclePharm Corporation. However, JJ, MM, and NL were not employees at the time of data collection, and the remaining authors have no financial interests concerning the outcome of this investigation. Additionally, this publication should not be viewed as endorsement by the investigators, the United States Sports Academy, the University of Northern Colorado, Metropolitan State University of Denver, the University of Nebraska, or MusclePharm Corporation.

## Authors’ contributions

CT, JJ, PF, LC, MM, JS, NL, MK, and JM contributed to conception of experimental design, drafting of the manuscript, and interpretation of data. CT, PF, LC, JS, SO, CM, and NL participated in data collection. Additionally, all authors read and approved the final manuscript.
